# High Individual Heterogeneity of Neutralizing Activities against the Original Strain and Nine Different Variants of SARS-CoV-2

**DOI:** 10.3390/v13112177

**Published:** 2021-10-28

**Authors:** Rita Jaafar, Celine Boschi, Sarah Aherfi, Audrey Bancod, Marion Le Bideau, Sophie Edouard, Philippe Colson, Henri Chahinian, Didier Raoult, Nouara Yahi, Jacques Fantini, Bernard La Scola

**Affiliations:** 1 Méditerranée Infection, Institut Hospitalier Universitaire, 13005 Marseille, France; ritajaafar01@gmail.com (R.J.); Celine.BOSCHI@ap-hm.fr (C.B.); sarah.aherfi@ap-hm.fr (S.A.); audrey.bancod@univ-amu.fr (A.B.); Marion.LE-BIDEAU@ap-hm.fr (M.L.B.); sophie.edouard@univ-amu.fr (S.E.); philippe.COLSON@univ-amu.fr (P.C.); didier.raoult@univ-amu.fr (D.R.); 2 Microbes, Evolution, Phylogeny and Infection (MEPHI), Institut de Recherche pour le Développement (IRD), 13005 Marseille, France; 3 Unite UNIS, Aix-Marseille Université, 13010 Marseille, France; henrichahinian@gmail.com (H.C.); nouara.yahi@univ-amu.fr (N.Y.); jm.fantini@gmail.com (J.F.); 4 Federation de Microbiologie, Assistance Publique-Hôpitaux de Marseille (AP-HM), 13005 Marseille, France; 5 Faculté de Médecine, Institut National de la Santé et de la Recherche Médicale (INSERM UMR_S 1072), 13015 Marseille, France

**Keywords:** SARS CoV2, variants, antibodies, COVID-19, vaccine, heterogeneity

## Abstract

Background: Since the beginning of the COVID-19 pandemic, several SARS-CoV-2 variants have sequentially emerged. In France, most cases were due to spike D641G-harbouring viruses that descended initially from the Wuhan strain, then by the variant of B.1.160 lineage we called Marseille-4 since the summer of 2020, which was followed by the Alpha and Beta variants in early 2021, then the Delta variant currently. Methods: We determined the neutralising antibody (nAb) titres in sera from convalescent individuals previously infected by these four major local variants and from vaccine recipients to the original Wuhan strain and nine variants, including two recent circulating Delta isolates. Results: The results show high inter-individual heterogeneity in nAbs, especially according to the variant tested. The major variations among nAbs are based on the genotype responsible for the infection. Patients previously infected with the beta and B.1.160 variants had the lowest nAb titres. We show that this heterogeneity is well explained by spike protein mutants modelling using in silico approaches. The highest titres were observed in individuals vaccinated with the Pfizer/BioNTech COVID-19 vaccine, even against the delta variant. Conclusions: Immunity acquired naturally after infection is highly dependent on the infecting variant, and, unexpectedly, mRNA-based vaccine efficacy was shown to be often better than natural immunity in eliciting neutralising antibodies.

## 1. Introduction

SARS-CoV-2 is the seventh member of the *Coronaviridae* family that infects humans and causes COVID-19. As of October 6, 2021, more than 230 million infections with approximately 4.8 million deaths have been recorded [1]. In France, despite declining numbers of COVID-19 patients in intensive care units, controlling and preventing the spread of the virus remains crucial [2]. Across the world, vaccines have been developed and commercialised, and the implementation of vaccination strategies and policies has become a priority.

The practice of immunisation dates back hundreds of years. In 1798, the first smallpox vaccine was developed, and since then, multiple vaccines have been developed and are available. However, during the last three decades, molecular genetics and its emphasis in immunology, microbiology, and genomics have had a great impact on the field of vaccinology [3]. This has led to the development of new vaccine types and delivery systems, such as DNA, RNA, and viral vectors, as well as an inactivated or even live attenuated forms of viral or bacterial pathogens [4]. As of 12 September 2021, a total of 671,767,335 doses of COVID-19 vaccines have been provided to countries in the European Union and the European Economic Area (EU/EEA) [5]. Of these vaccines, 69% of all doses distributed to EU/EEA countries were with the Pfizer/BioNTech (BNT162b2) COVID-19 Vaccine, followed by AZD1222 COVID-19 Vaccine by AstraZeneca (14%), Spikevax COVID-19 Vaccine by Moderna (12%), and the COVID-19 Vaccine by Janssen (3%). Since the start of COVID-19 vaccine deployment in the EU/EEA in December 2020, the cumulative vaccine uptake in the adult population (aged 18 years and older) in the EU/EEA has progressed as reported in 30 countries, reaching 78.1% for at least one vaccine dose and 71.6% for the full vaccination course [5]. The remaining questions are whether the antibodies generated after vaccination and after natural infection confer long-lasting immunity and whether rapidly evolving mutants, especially those with spike protein mutations, modify the vaccines’ effectiveness.

Four new variants that have rapidly become dominant in the mentioned countries have garnered concerns: the Alpha (PANGO Lineage: B.1.1.7) variant that was identified in the United Kingdom (UK), the Beta (PANGO lineage: B.1.351) variant that was first detected in South Africa, the Gamma (PANGO lineage: P.1) variant that first spread in Brazil, and the Delta (PANGO lineage: B.1.617.2) variant that was first identified in India. These variants that emerged between late 2020 and the beginning of 2021 are now classified by the CDC as “variants of concerns” (VOCs) due to their transmissibility, mortality, and immunogenicity characteristics. However, other variants, such as the Marseille-4/B.1.160 variant, were responsible for a large number of cases and associated deaths such as in France [6]. Although policies to prevent the spread of SARS-CoV-2 variants are implemented, the high mutation rate and rapid emergence of variants of this RNA virus highlight the importance of vigilance with regard to the genomic surveillance for the early identification of future variants. Eliciting broadly neutralising activity against current and potential future variants is now considered a must to evaluate vaccine efficiency and to prevent reinfections.

In this cohort, we aimed to analyse the reactivity by seroneutralisation tests towards 10 different SARS-CoV-2 strains in sera from patients assumed to be immune to SARS-CoV-2 either from a previous natural infection with this virus or from immunisation by two injections of the SARS-CoV-2 vaccine. These data were interpreted in light of a comparative structural analysis of the spike proteins expressed by the different SARS-CoV-2 strains studied.

## 2. Materials and Methods

### 2.1. Serum Samples and Human Monoclonal Antibodies

A total of 55 human serum samples were included as part of a sero-epidemiological study that is being performed in our laboratory, and the patients’ sera included 42 sera obtained from convalescent patients within 3 weeks to 5 months after a documented COVID-19 infection (Tables S1 and S2). Eleven patients were infected by spike D614G-harbouring B lineage strains that spread during the first wave of COVID-19 infections in France, 9 were infected by the Marseille-4/B.1.160 variant, 10 were infected by the Alpha variant, and 12 were infected by the Beta/B.1.351.2 variant. Direct genotyping from respiratory samples was performed under previously described conditions [7]. Along with these sera, 13 sera from vaccinated individuals were also selected. Eleven of these individuals received 2 shots of the Pfizer/BioNTech COVID-19 vaccine (BNT162b2), and two received 2 shots of the AZD1222 COVID-19 vaccine. These vaccinated individuals were sampled within 2 to 12 weeks after their second shot. For the human monoclonal antibody, we selected LY-CoV555 to be tested along with the serum samples in the microneutralisation test (MNT) and used it as our positive control for the tested antibodies in the established assays.

### 2.2. Ethical Statement

This study was approved by the Ethics Committee of the IHU Mediterranée Infection under the number 2021–011 on 19 April 2021. The serum samples were collected for diagnostic purposes and were reused for the MNT anonymously. According to French law (loi Jardé), anonymous retrospective studies do not require institutional review board approval nor informed consent.

### 2.3. Serological IgG Test

Specific anti-SARS-CoV-2 IgG antibodies were detected by the Liaison XL automated chemiluminescent immunoassay (CLIA) (Diasorin Inc., Saluggia, Italy) according to the manufacturer’s recommendations. This test uses magnetic beads coated with antigens derived from subunits S1 and S2 of the viral spike protein.

### 2.4. Cell Line Preparation and Subculturing Procedure

Vero E6 cells (ATCC-CRL-1586) were propagated and cultured in minimal essential medium (MEM, Gibco, USA) supplemented with 2 mM L-glutamine and 10% foetal bovine serum (FBS) at 37 °C in a 5% humidified incubator. Ninety-six-well plates of Vero E6 cells were prepared for the neutralisation tests of SARS-CoV-2 in MEM growth medium supplemented with glutamine and 4% FBS.

### 2.5. Viral Strains

The viruses used in our study correspond to the strains isolated at our laboratory, IHU-Méditerranée Infection, as a part of routine virology work. Viral strains were isolated in cell culture from patients’ clinical samples under previously described conditions and then frozen at −80 °C for further use [8]. All strains were confirmed as SARS-CoV-2 and genotyped by whole genome next-generation sequencing as previously described [5] (Table S3). For the microneutralisation test (MNT), virus production was performed by thawing the previously conserved virus suspension and reinoculating in a previously prepared 12-well Vero E6 cell plate at a density of 4 × 10^5^ cells/mL. After 48 h, the virus suspension was harvested and quantified by real-time reverse-transcription (RT)-PCR (qPCR) and TCID50 determination.

### 2.6. Micro-Neutralisation Test (MNT)

Our study was based on a cytopathic effect (CPE)-based MNT. Each serum sample was assayed for neutralisation against the 10 SARS-CoV-2 strains. Sera were heat-inactivated at 56 °C for 1 h. Twofold serial dilutions from 1:5 to 1:640 were prepared and then mixed with each of the 10 tested viral strains that had been previously quantified by qPCR and normalised to a cycle threshold value (Ct) of 25 through dilution of the viral stock with culture medium (MEM, supplemented with 4% FBS and 2 mM glutamine). This normalisation was verified by reading the TCID50 at 5 days and corresponded to 4.35 ± 0.23 log_10_ virus/mL for all strains. The serum/virus mixture was incubated for 1 h at 37 °C in a humidified atmosphere with 5% CO_2_. After incubation, 100 µL of cell culture medium was removed, and 100 µL of the mixture at each dilution was added in quadruplicate to a 96-well cell plate containing a subconfluently Vero E6 cell monolayer. The plates were incubated for 5 days at 37 °C in a humidified atmosphere with 5% CO_2_. The same procedure was established for the human monoclonal antibody, except for the antibody dilution that corresponded in 1:5 serial dilutions going from a concentration of 3500 μg/mL to 0.0089 μg/mL of LY-CoV555. After 3–5 days of incubation, the plates were inspected by an inverted optical microscope. On the fifth day, the highest serum dilution that protected at least 50% of cells from CPE was taken as the neutralisation titre.

### 2.7. Statistical Tests

We performed a statistical analysis using GraphPad Prism v9.0.0 (GraphPad Software, LaJolla, CA, USA) using an analysis of variance (ANOVA), followed by Tukey’s multiple comparisons test. *p*-values ≤ 0.05 were considered as significant.

### 2.8. Computational Methods

The spike protein mutants were modelled using in silico approaches. As the mutations are localised in two different domains of the spike, namely, the N-terminal domain (NTD) and the receptor binding domain (RBD), separate models were generated for each domain. The atomic coordinates of the RBD bound to LY-CoV 555 neutralising antibody (nAb) were retrieved from PDB file 7KMG [9], and the structure of the NTD bound to the 4A8 nAb was retrieved from PDB file 7C2 L [10]. Minimised structures of the RBD and NTD of each variant were obtained by introducing appropriate mutations and/or deletions in the initial PDB files. Energy minimisations of the variants were performed with the Polak–Ribière conjugate gradient algorithm with the Bio-CHARMM force field in Hyperchem [11] using a maximum of 3 × 10^5^ steps and a root mean square (RMS) gradient of 0.01 kcal. Å^−1^.mol^-1^ as the convergence condition. The variant domain was then merged with the corresponding nAb (LY-CoV555 for the RBD, 4A8 for the NTD) using the initial coordinates of the 7KMG and 7C2 L pdb files, and the whole system was succumbed to a new series of energy minimisations. The energy of interaction of each complex was calculated with a Molegro molecular viewer as previously described [12,13].

## 3. Results

### 3.1. Convalescent Plasma

**Figure 1:**
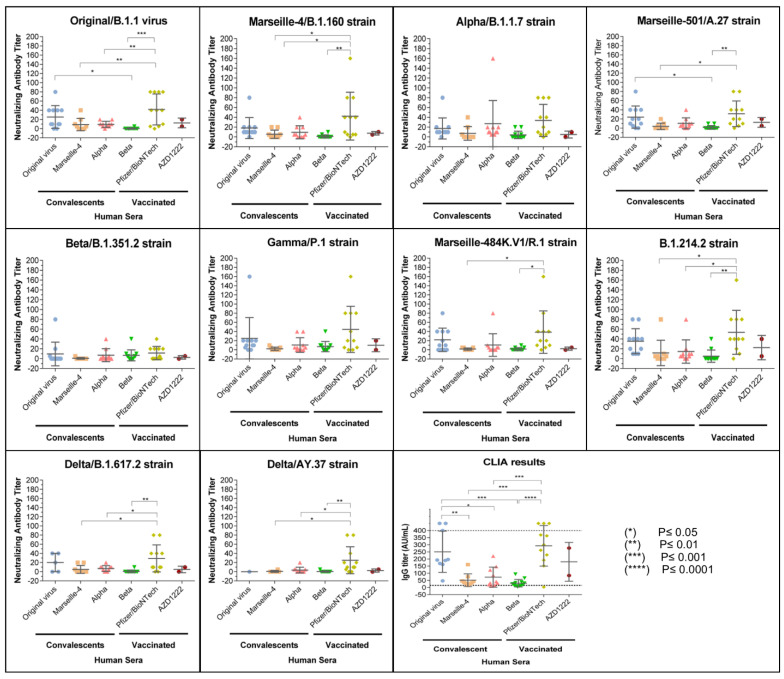
Figure showing the neutralising response against the original virus (Original/B.1.1) and the 9 SARS-CoV-2 variants in convalescent patients and in vaccinated participants. The *Y*-axis represents the neutralising antibody titres obtained by MNT, and the *X*-axis represents the corresponding group of tested human sera. Data are shown as the mean and standard error of the mean (SEM). Solid lines represent the geometric mean titre, and the whiskers show the 95% confidence interval. Each scatter represents one serum. Scatter symbols and colours are attributed on the basis of the sera group. Serology results are shown for the convalescent patients and for the vaccinated patients. For seroneutralisation, the 10 quadrants correspond to one different tested strain each. The last quadrant shows the IgG titres in AU/mL as obtained by chemiluminescent immunoassay (CLIA) for each serum group. IgG titres > 400 are represented in the graph above the maximum threshold of detection (400 AU/mL). The same statistical significance was obtained by excluding these nonquantitative values (>400) from the ANOVA test. In all graphs, significance is represented by an asterisk for *p* ≤ 0.05. The absence of an asterisk means no significant variance was detected between the groups.

**Figure 2:**
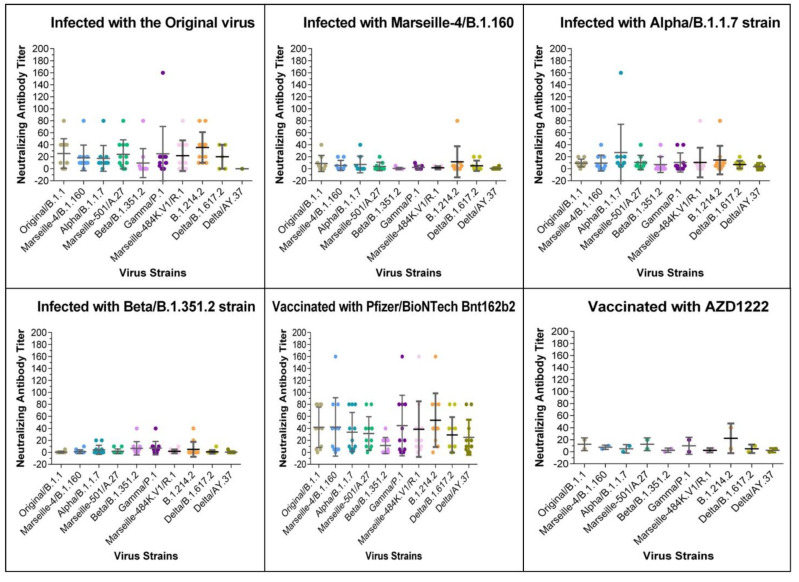
Figure showing the neutralising response of each serum group against the original virus (Original/B.1.1) and the 9 SARS-CoV-2 variants in convalescent patients and in vaccinated participants. The *Y*-axis represents the neutralising antibody titres obtained by MNT, and the *X*-axis consists of the different tested SARS-CoV-2 strains. Data are shown as the mean and standard error of the mean (SEM). Solid lines represent the geometric mean titre, and the whiskers show the 95% confidence interval. Each scatter represents one serum. Scatter’s symbols and colours are strain-specific. Each quadrant in the figure corresponds to one different sera group. In all graphs, significance is represented by an asterisk for *p* ≤ 0.05. Significance is represented as an asterisk for significant *p*-values. The absence of an asterisk means no significant variance was detected between groups.

The IgG titres provided by the CLIA test are shown in Figure 1 and Figure 2, and Table S4. According to the manufacturer, output results were considered positive for the presence of anti-SARS-CoV-2-IgG antibodies for values >15 AU/mL, negative for values <12 AU/mL, and as borderline for values between 12 and 15 AU/mL. Among convalescent COVID-19 patients tested by CLIA, 34/40 (85%) sera were IgG-positive, 2/40 (5%) (SA-1, SA-6) were considered borderline, and 4/40 (10%) were negative (Table S1). By arbitrarily classifying the patients with high titres of antibodies (IgG titres > 100), we found that the prevalence of the high titres decreased among patients infected by the original/B.1.1 genotype (8/9, 89%), the Alpha genotype (3/10, 30%), the Marseille-4/B.1.160 genotype (1/9, 11%), and the Beta genotype (0/12, 0%). Moreover, a significant proportion (3/12, 25%) of the patients infected with the Beta variant were negative for IgG by CLIA, and these samples represented 75% of all samples that tested negative.

For MNT, the observed titres were low, ranging from no seroneutralisation (<1/5) to 1/160 (Figure 1). The same was found for CLIA in terms of the reactivity of the different groups of convalescent sera. By arbitrarily classifying the patients with high IgG antibody titres ≥ 1/10 against the strain that the patients were infected with, we found that the decreasing order of the prevalence of titres were: the original/B.1.1 genotype (9/11, 82%), Alpha genotype (8/10, 80%), Marseille-4/B.1.160 genotype (2/9, 22%), and Beta genotype (3/12, 25%). The presence of neutralising antibodies in each group of patients against the variants, excluding those variants responsible for the patients’ infection, varied according to the variant tested. Without taking into account the sera of patients convalescing for the Beta variant that react nearly only to Beta and Gamma variants, at a 1/5 MSN titre, 14/30 (47%) of the samples reacted against the Original/B.1.1 genotype, 10/19 (53%) reacted against the Marseille-4/B.1.160 genotype, 13/20 (65%) reacted against the Alpha genotype, 20/30 (66%) reacted against the Marseille-501/A.27 variant, 18/30 (60%) reacted against the Gamma variant, 15/60 (50%) reacted against the Marseille-484K.V1/R.1 variant, 24/30 (80%) reacted against the B.1.214.2 variant that was first described in Belgium, and 12/60 (50%) reacted against the Delta variant. The Beta variant was the least recognised variant after excluding the variants that caused the infections, as only 8/30 (27%) had detectable seroneutralising antibodies against this variant.

### 3.2. Vaccinated Patients’ Sera

Regarding the vaccinated participants and their CLIA serology test results, both participants who received the two AZD122 injections were IgG-positive, and the majority (11/12) of the participants were vaccinated with two doses of the Pfizer/BioNTech vaccine (Figure 1 and Figure 2, Table S5). However, one patient (V-Pfizer-10) who showed no detectable reaction by the seroneutralisation tests, even against the original Original/B.1.1 strain, despite the patient previously receiving two doses of the Pfizer/BioNTech vaccine, was also negative for IgG in the CLIA. This patient was not immunocompromised but was an elderly patient (88 years old). The other patient with a low antibody titre (V-Pfizer 4) had a previous splenectomy.

The neutralising profiles of most patients who had the Pfizer/BioNTech vaccine also showed neutralisation gaps in the Beta variant (Figure 1 and Figure 2). Otherwise, these sera appeared to inhibit the in vitro CPE for 9 out of the 10 SARS-CoV-2 strains until the sera dilutions were 1:40 and 1:80. Both persons who received two shots of the AZD122 vaccine displayed limited to completely absent neutralisation on all the tested SARS-CoV-2 isolates. One Astra-2 serum showed a stronger reaction with the B.1.214.2 variant isolate. The results of individuals who received the Pfizer/BioNTech vaccine also showed heterogeneity in the neutralisation profiles, as some had much weaker antibody titres than those vaccinated and had very high IgG titres (>400 AU/mL). Of interest for the current period, 8/11 patients vaccinated by the Pfizer/BioNTech vaccine had MSN titres ≥ 1/10 against both of the Delta variant strains tested.

### 3.3. Human Monoclonal Antibody LY-CoV555

**Figure 3:**
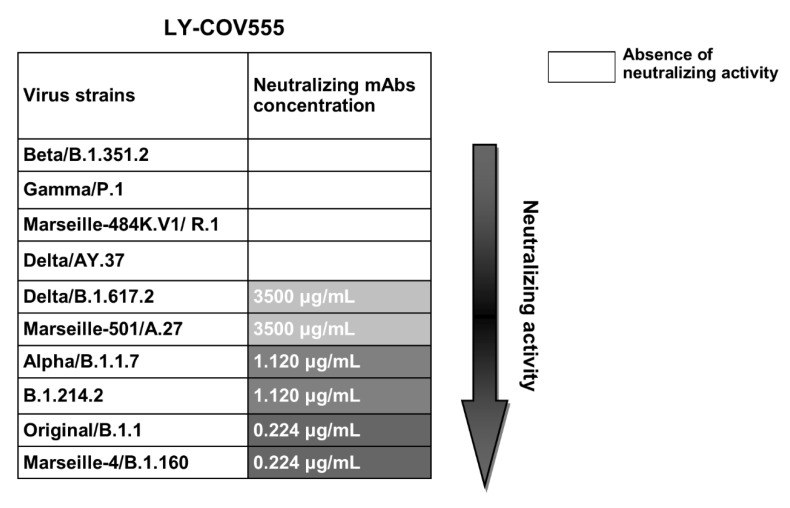
Figure showing a scheme of the neutralising activity results for the monoclonal antibody bamlanivimab. Table showing the neutralising activity of LY-CoV555 against the 10 tested strains. Neutralising concentrations are represented by grey colour gradient cases and are displayed in μg/mL units. Darker grey colours reflect higher neutralisation activity.

We assayed the neutralising activity of the commercial monoclonal antibody bamlanivimab (LY-CoV555) at an initial concentration of 35 mg/mL, and the results of the neutralisation activity of this tested mAb are summarised in the first row in Figure 3. LY-CoV555 significantly neutralised the Original/B.1.1 strain and the Marseille-4/B.1.160 variant (neutralising titre of 0.224 μg/mL), and less significantly neutralised the Alpha and B.1.214.2 variants (neutralising titre of 1.12 μg/mL). Additionally, it had almost no neutralising activity on the Marseille-501/A.27 and Delta variants (very low neutralising titre of 3500 μg/mL). Moreover, the Beta and Marseille-484K.V1/R.1 variants were profoundly resistant to neutralisation by bamlanivimab (LY-CoV555).

### 3.4. Molecular Mechanisms of the Neutralisation Escape of SARS-CoV-2 Variants

Most neutralising antibodies (nAbs) against SARS-CoV-2 are directed against the RBD and the NTD of the spike protein. As references, we used the LY-CoV 555 nAb (bamlanivimab) and the 4A8 nAb, which recognise the principal neutralisation determinants of the RBD and the NTD, respectively.

**Figure 4:**
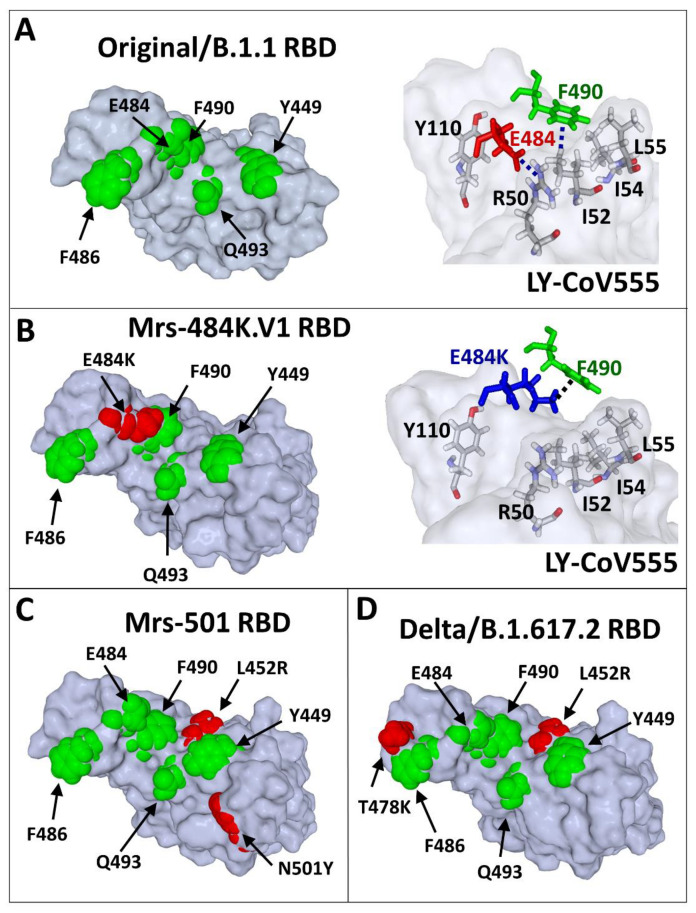
Scheme showing the molecular mechanisms of nAb escape in the RBD of SARS-CoV-2 variants. (**A**) The epitope recognised by the LY-CoV555 nAb (pdb file #7KMG) consists of several amino acid (coloured in green) residues distributed on the surface of the RBD. The anionic carboxylic group of E484 interacts with the cationic charge of R50 (heavy chain of LY-CoV555 nAb) through an electrostatic bridge. The aromatic ring of Y490 interacts with a methyl group of I52 (heavy chain of LY-CoV555 nAb) by a CH–π interaction, which is reinforced by vicinal apolar amino acid residues (I54 and I55). (**B**) In the Marseille-484K.V1/R.1 variant, E484 (in red in the left panel) is mutated in E484K (in blue in the right panel). The consequence of this mutation is a shift of the side chain of E484K whose cationic group (which replaces the negative charge of E484) now forms a cation–π bond with the aromatic ring of F490. In this new context, neither E484K nor F490 can still interact with the LY-CoV555 nAb. Indeed, R50, I52, I54, and L55 of the heavy chain of the antibody are no longer involved in RBD recognition. (**C**) Mutational pattern of the Marseille-501/A.27 variant (L452R/N501Y). (**D**) Mutational pattern of the India_1 variant (L452R/T478K). The same molecular modelling method was applied to all variants (**B**–**D**) after introducing the mutations in the reference Original/B.1.1–nAb complex (PDB file #7KMG), followed by energy minimisation of the RBD and simulations of the binding reaction.

The E484K substitution (Glu → Lys substitution) in the Marseille-484K.V1/R.1 variant induces a dramatic rearrangement of the RBD surface that results in a complete lack of interaction with the bamlanivimab nAb (Figure 4A,B). This molecular mechanism explains the dramatic decrease in the affinity (85%) of the bamlanivimab nAb for the RBD of the Marseille-484K.V1/R.1 variant (Table 1). A similar mechanism also accounts for all variants that display the E484K mutation, including the Beta (70% decrease) and Gamma (67% decrease) variants (Table 1).

**Figure 5:**
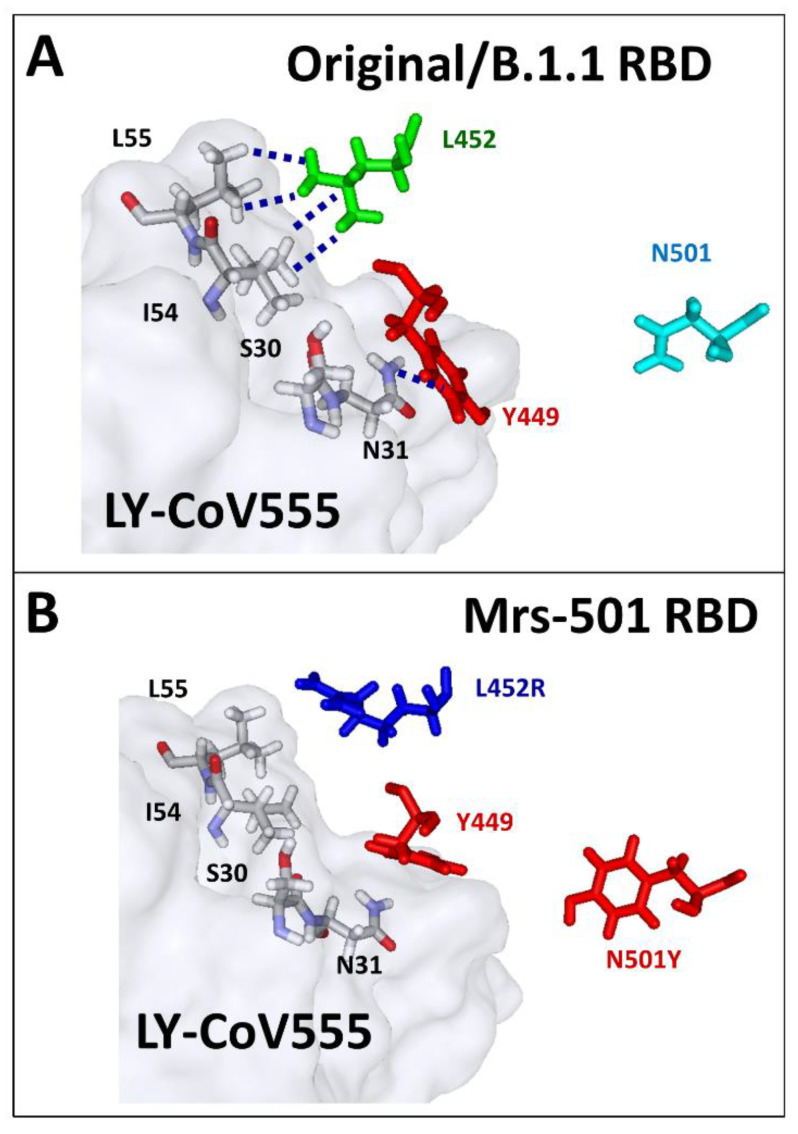
Figure showing the molecular mechanism of nAb escape by the RBD of the Marseille-501/A.27 (Mrs-501) variant. (**A**) In the Original/B.1.1 RBD (PDB file #7KMG), the heavy chain of the LY-CoV 555 nAb interacts with the side chains of L452 (Van der Waals network) and Y449 (NH–π). (**B**) In the Marseille-501/A.27 variant, the mutant R452 is displaced out of the Van der Waals network, which reorients Y449 so that the NH–π interaction with N-31 is no longer possible. The aromatic ring of Y501 comes closer to Y449, which definitely prevents any contact with the heavy chain of the nAb.

The L452R substitution is present in both the Marseille-501/A.27 (Figure 4C) and the Delta (Figure 4D) variants, yet in a distinct mutational context. In the case of the Marseille-501/A.27 variant, the L452R mutation is associated with N501Y. The loss affinity of bamlanivimab’s nAb for this variant was estimated to be 76% (Table 1). The molecular mechanism of this effect could be attributed to a reorientation of the cationic side chain of R452 (compared to L452), which takes Y449 away from the antibody heavy chain residue N31 (Figure 5A,B).

**Figure 6:**
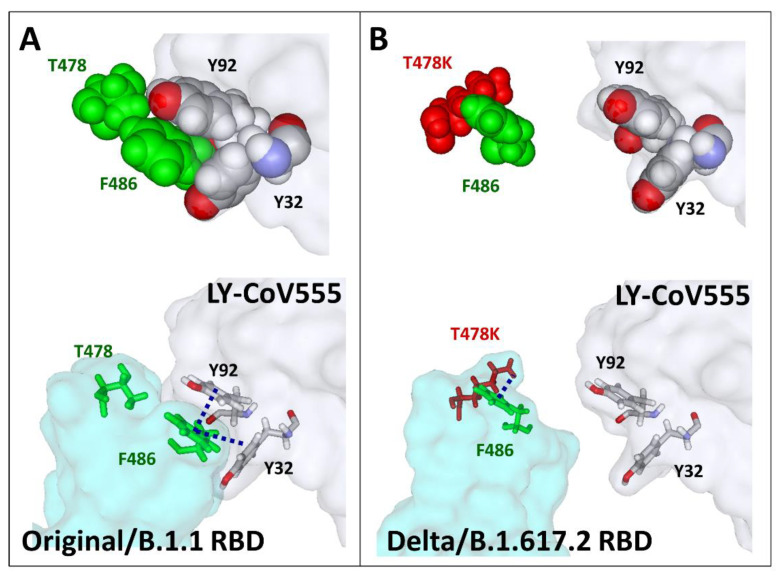
Figure showing the molecular mechanism of nAb escape by the RBD of the Delta/B.1.617.2 variant. (**A**) A π–π aromatic cluster is involved in the recognition of the RBD (Original/B.1.1 strain) by the LY-CoV555 nAb (pdb file #7KMG). Y32 and Y92 of the light chain of the antibody clamp the aromatic ring of RBD residue F486. This cluster is stabilised by a CH–π interaction between the methyl groups of T478 and F486. It should be noted that this interaction is important to functionally orient and wedge the side chain of F486 between Y32 and Y92. (**B**) In the Delta/B.1.617.2 variant, the mutation T478K prevents the formation of this network by forming a cation–π interaction between the cationic group of this residue and the aromatic ring of F486. This new bond reorients the side chain of F486 towards the RBD surface, thereby preventing any possibility of association with the antibody. The models are shown in sphere (upper panels) or stick (lower panels) representations.

The case of the Delta variant is more puzzling, since, in this case, the substitution L452R is associated with T478K instead of N501Y. As shown in Figure 6A, in the Original/B.1.1 strain, T478 is close to F486, a key amino acid controlling bamlanivimab recognition. Indeed, the methyl group of T478 points in the direction of the aromatic ring of F486, which allows the formation of a cluster of π–π interactions with Y32 and Y92 of the light chain of the antibody. The clamp of Y32 and Y92 is particularly visible when the amino acid atoms are represented in spheres (Figure 6A, upper panel). When T478 is substituted by K478 (T478K substitution), F486 is attracted by the cationic group of K478, preventing any contact with the aromatic amino acids Y32 and Y92 of the antibody (Figure 6B). This mechanism largely contributes to the 72% loss of affinity of bamlanivimab for the RBD of the Delta variants (Table 1).

**Figure 7:**
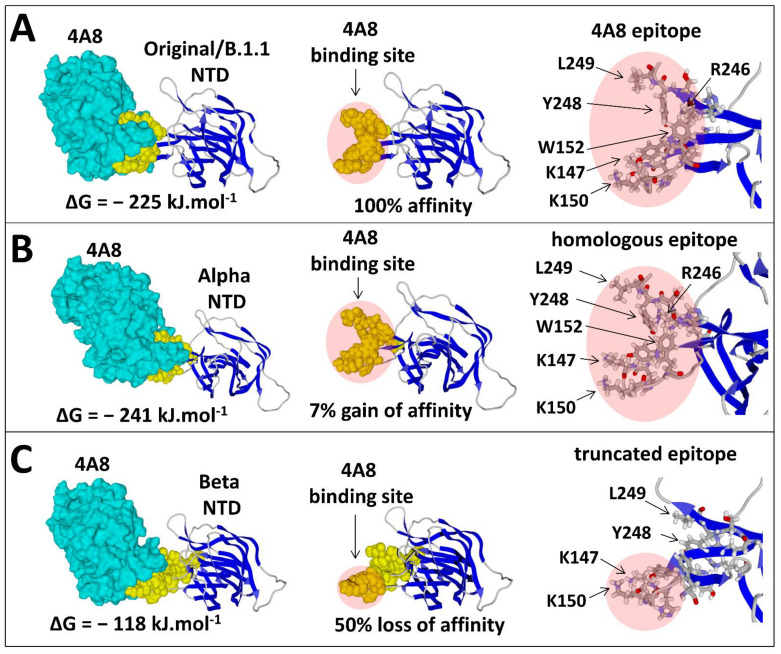
Figure showing the variability of the main neutralising epitope in the NTD among virus strains. (**A**) Molecular mechanism of NTD recognition (Original/B.1.1 strain) by the 4A8 nAb. The NTD–nAb complex (pdb file #7C2 L) has a global affinity of −225 kJ/mol^−1^. The antibody clamps two distal zones of the NTD (the N3 loop with amino acid residues K147, K150, and W152) and the N5 loop (R246, Y248, and L249), which together form the main neutralising epitope of the NTD. (**B**) The NTD of the Alpha variant retains this crescent-shaped structure, which displays a slightly higher affinity (+ 7% compared with the Original/B.1.1 strain) for the 4A8 nAb due to the repositioning of the amino acids of the N3 loop. (**C**) In the case of the Betavariant, only the N3 loop part of the epitope is conserved, and therefore that the affinity for the 4A8 nAb is decreased by 50%. Such a truncated epitope may elicit a poor antibody response, consistent with seroneutralisation data.

Finally, we evaluated the impact of mutations in the NTD on antibody recognition (Table 1). Interestingly, some mutational patterns did not seem to decrease the affinity of the 4A8 nAb for the NTD, and in some cases, the affinity was even slightly increased, as shown for the Marseille-484K.V1/R1 and Alpha variants (Table 1). In other cases, a significant decrease in the antibody affinity, compatible with the neutralisation escape, was calculated and ranged from 64% for the Gamma variant to 47% for the Beta variant (Table 1). At the opposite end of the scale, the affinity of 4A8 for the Alpha variant was slightly increased (−241 kJ.mol^−1^ vs. −225 kJ.mol^−1^ for the Original/B.1.1 strain). A detailed analysis of the 4A8 epitope provided a molecular explanation for such a range of effects (Figure 7). This epitope is divided into two prominent and flexible regions of the NTD, the N3 and N5 loops, which adopt a crescent-like shape recognised by the antibody (Figure 7A). Key residues involved in 4A8 binding belong either to the N3 loop (K147, K150, and W152) or to the N5 loop (R246, Y248, and L249). Inasmuch as both loops are accessible at the NTD surface, the 4A8 antibody can bind to the NTD, as shown for the Origjnal/B strain (Figure 7A) and the Alpha variant (Figure 7B). In the case of the Beta variant, the only part of the epitope preserved from this dramatic reorganisation of the NTD is the tip of the N3 loop harbouring K147 and K150, which may explain the residual affinity of some anti-NTD nAbs (such as those elicited by vaccination) for this variant (Figure 7C).

**Figure 8:**
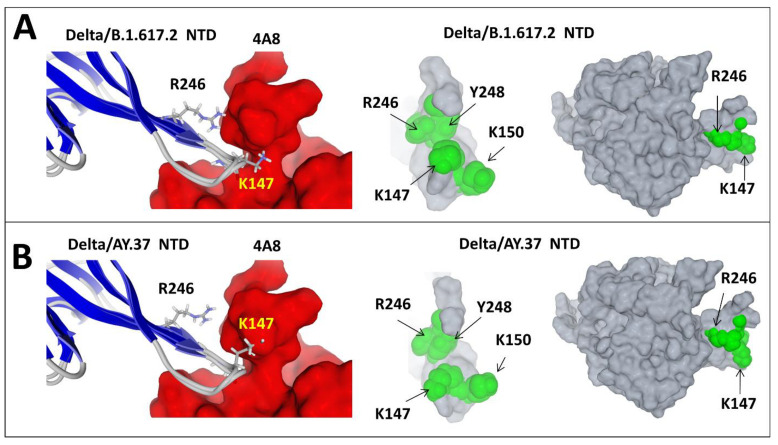
Figure showing the intralineage variability of the main neutralising epitope in the NTD: the case of India variants. (**A**) Delta/B.1.617.2 variant. (**B**) Delta/AY.37 variant. The left panels in (**A**) and (**B**) show the positions of residues K147 and R246 that face the antibody. The superposition of the secondary structure shows that the conformational changes between these variants chiefly involve the amino acid side chain orientation. In the case of Delta/B.1.617.2, both K147 and R246 interact with the antibody, whereas in the case of Delta/AY.37, R246 moves away from the antibody, while K147 gets closer, resulting in decreased binding to avoid steric clash. The middle panels show the NTD surface as “seen” by the antibody. The right panels show the subtle rearrangements of the NTD structure of both India variants. The main change concerns the orientation of K147.

However, this truncated epitope may lose most of its immunogenicity. Thus, patients infected by the Original/B.1.1 or the Alpha strains may elicit nAbs against several variants, including the Beta, but the reverse is not true, as sera from patients infected by the Beta variant have poor neutralising activities. Subtle conformational changes in the NTD affecting the relative orientations of the K147 and R246 side chains were consistent with the slightly decreased affinity of anti-NTD nAbs for the Delta/AY.37 variant vs. the Delta/B.1.617.2 variant (Figure 8). Since both variants have the same RBD but display distinct mutational patterns in the NTDs, these data underscored the importance of the NTD as a key neutralising determinant of SARS-CoV-2.

## 4. Discussion

In this study, we report a serological investigation using a CPE-based microneutralisation assay of anti-SARS-CoV-2 antibodies and tested 10 different strains of this virus including the original strain that initially spread and 9 variants. Seroneutralisation assays are always the gold standard for in vitro assays. This technique performed with replication-competent coronaviruses seems to have an epidemiological potential to detect the presence or absence of neutralising antibodies against the newly emerging SARS-CoV-2 variants. This technique is clearly superior to pseudoviral systems that do not reflect a real viral infection since they only mimic the entry step of the virus’ life cycle and have serious limitations related to the usage of unnatural core proteins [14]. We managed to obtain convalescent plasma samples from 42 patients at least 3 weeks after a documented SARS-CoV-2 infection. As illustrated in Figure 2, the overall antibody response was divided into four groups, each group representing a period of infection.

A remarkable neutralisation pattern with high antibody levels was seen in convalescent patients recovered from the original Original/B.1.1 strains (PANGO Lineage B.1.1), as determined by CLIA. Afterwards, the humoral response was also detected in patients previously infected with the Alpha variant between March and April 2021, and these patients had moderate levels of IgG following the infection. Additionally, antibody titres were shown to be reduced to low levels in patients previously infected with the Marseille-4/B.1.160 variant, and very low IgG levels were detected in the plasma of patients recovered from infection with the Beta variant.

Two groups of patient antibodies seem to significantly recognise the strain causing the infection, as seen in the case of Original/B.1.1 strain patients and Alpha variant patients. This was not seen in patients who had recovered from an infection with the Marseille-4/B.1.160 and Beta variant strains. However, some positive IgG sera in the patients infected with the Beta variant contained antibodies that are able to neutralise this variant, albeit poorly (neutralisation titres ranging mostly from 1:5 and 1:10). This variant is particularly interesting because it displays both single point mutations and deletions, which induce a global reorganisation of the NTD. However, the tip of the N3 loop harbouring K147 and K150 was only marginally affected by this reorganisation, which may explain the residual affinity of some nAbs for the NTD.

Regarding the global humoral response, our data highlight a strong variability in the antibody levels and in the neutralisation profiles. The reason behind this “interindividual heterogeneity” is not yet clear. However, multiple studies have shown a sort of positive correlation between the serum neutralising capacity and disease severity, highlighting the highly heterogeneous nature of nAb responses against the SARS-CoV-2 spike protein [15,16]. In response to the question of what the immunity and protection levels are following a natural infection against the currently circulating variants, especially the VOCs, we also studied the reactivity between nAbs and nine different variant isolates. Our results show that infection with the Marseille-4/B.1.160 and the Beta variants did not confer humoral protection against the majority of the circulating strains. However, a relative but significant immunity was observed for those patients who recovered from the original strain that circulated between March and June 2020, as well as the patients who had the Alpha variant in reactions against all of the strains except for the Beta strain. Our data suggest that this variant strain (Beta variant) is ultimately resistant to the activity of nAbs. This in vitro resistance correlates with the first reinfection case reported in France by February 2021 by the Beta SARS-CoV-2 VOC (beta, V2), which caused a severe case of COVID-19 4 months after the first mild infection [17]. Our findings also correlate with another in vitro assay confirming that the Beta variant may escape the neutralising antibody response elicited by prior natural infection with a half maximal inhibitory concentration (IC50) 6 to 200 times higher than that of the virus in the first wave of the pandemic [18]. A potential hypothesis may have arisen concerning intrahost evolution in some individuals with sustained viral replication where the genetic diversity from a continuous turnover of dominant viral species may have resulted from differential selective pressures [19,20]. As the receptor binding motif (RBM) is considered the main functional motif that forms the interface with the human ACE2 (hACE2) receptor, multiple studies have shown that the corresponding epitope mutation centred around E484 led to various amino acid changes and strongly affected plasma antibody neutralisation [21,22,23,24,25]. This can be seen for the Gamma and Marseille-484K.V1/R.1 variants harbouring the same spike key mutation (E484K), as they show a similar but potential immune escape (with low nAb recognition). The last combination of mutations that are currently spreading worldwide was previously identified as the new Delta variant that first emerged in India in October 2020 and spread further in many countries. Due to its key spike protein mutations (L452R and T478K), the Delta variant may induce an immune evasion [26], similar to the B.1.351.2, P.1, and R.1 variants. As shown in Table 1, the Delta variant strain seems to be resistant to recognition by the LY-CoV555 (bamlanivimab) monoclonal antibody that we tested in our study. These findings are similar to those recently described in a new study published in July 2021 [27]. The article showed that there was a reduced sensitivity of the Delta variant to the sera from convalescent patients and vaccinated individuals, and our results on the sera of patients previously infected with the original Original/B.1.1 strain Alpha and the Marseille-4/B.1.160 variants also had less reactivity towards this variant.

In our study, we finally tested the mAb named LY-CoV555, which was authorised for emergency use by the FDA. By April 16th, 2021, the FDA revoked the emergency use authorisation (EUA) [28] that allowed the investigational monoclonal therapy by bamlanivimab to be used [29]. On the basis of new data and ongoing analyses in addition to the increase in SARS-CoV-2 viral variants that are shown to be resistant to mAbs, therapy with bamlanivimab alone [30] has resulted in an increased risk for treatment failure. Although our data confirm these conclusions, as we see in Table 1, bamlanivimab neutralises only the Alpha variant, Marseille-4/B.1.160, the B.1.214.2 variant, and the original Original/B.1.1 strain. For other VOCs, no neutralising activity of these mAbs was observed.

Our molecular modelling data are in complete agreement with this finding (Table 1). The variants that are neutralised by bamlanivimab are well recognised by this antibody, with ∆G values ranging from −195 to −245 kJ.mol^−1^, which is close to the ∆G of the reference Original/B.1.1 strain nAb complex (−244 kJ.mol^−1^). In contrast, variants that resist bamlanivimab seroneutralisation (Marseille-484K.V1/R.1, Marseille-501/A.27, Gamma, both Delta, and Beta) have very low affinity for the nAb, with values of ∆G ranging from −36 to −80 kJ.mol^−1^. The analysis of the NTD–nAb complex of each variant confirmed this classification, although the NTD appeared to display interesting features. In general, the loss of affinity of the RBD for nAbs was associated with a similar loss of affinity of the NTD for its own nAbs. The only exception to this rule was the Marseille-484K.V1/R.1 variant because it did not display any mutation in the NTD. In all other cases, there was a good correlation between the neutralisation escape of the RBD and of the NTD. However, residual ∆G values were globally higher for variant NTDs than for variant RBDs. In fact, the affinity of variant NTDs was decreased by 64% at maximum (Gamma variant), compared to 85% in the case of the RBD (Marseille-484K.V1/R.1 variant). For the Beta variant, the loss of affinity of the anti-NTD nAb was estimated to be 48% (Table 1). This finding is in good agreement with the seroneutralisation data, which showed that 42.9% of fully vaccinated individuals could not neutralise the Beta variant [31].

Structural analysis of the 4A8 epitope revealed that it is partially formed by a flexible loop of the NTD (the N3 loop) that may remain accessible for the nAb even in the presence of multiple mutations and/or deletions in the NTD (Figure 7). Thus, the sensitivity of a given variant to seroneutralisation may depend on the relative balance between anti-RBD and anti-NTD nAbs in the patients’ sera. This balance may in fact explain the discrepancy in seroneutralisation studies that concluded that SARS-CoV-2 variants could be either partially or totally resistant to nAbs [24,32]. Initially, it was assumed that most anti-SARS-CoV-2 nAbs were directed against the RBD [33] []. A more recent analysis [34] challenged this view and concluded that the prevalence of anti-NTD neutralising antibodies was higher than the anti-RBD nAbs in convalescent subjects. Indeed, more than 80% of the immunological response lies outside the RBD. Combined with our modelling study, these data suggest that the heterogeneity of SARS-CoV-2 seroneutralisation mostly reflects the neutralising activity of anti-NTD antibodies. The lower the anti-NTD nAb titre, the higher the immunological escape. It is also important to note that several variants (e.g., Beta variant) have a lower accessibility of the N5 loop to nAbs (Figure 7), and therefore any therapeutic anti-NTD monoclonal antibody directed against this loop may be of limited use [35].

Structural analysis of the SARS-CoV-2 spike protein in conjunction with nAbs and molecular modelling studies of variants can help determine which variants of interest (VOIs) may become variants of concern (VOCs). This variant status can be estimated in real time from genome sequence data by calculating the transmissibility (T-index) [13] and, for the first time, the immune escape (I-index) capabilities. This I-index takes into account the impact of mutations in the RBD and of mutations/deletions in the NTD on the free energy variation (∆G) of each nAb–spike complex (Table 1). It perfectly separated the variants that escape the binding of antibodies, and by introducing an optimised I-index, we were able to to perfectly match it with the reactivity observed in the patients and thus predict the capability to escape antibodies of any upcoming SARS-CoV-2 strain.

The results from phase III clinical trials in the United Kingdom revealed that the BNT162b2 and AZD1222 vaccines were highly effective when using a two-shot protocol with a target interval of three and four weeks, respectively, between doses [36]. In addition to immunity following natural infection, we were interested in studying the acquired immunity following vaccination. In Europe and especially in France, vaccination and immunisation will now be available for all individuals that are older than 18 years of age [37]. Two doses of the vaccines are required to achieve adequate immunisation against COVID-19. Our data show that patients vaccinated with the mRNA-based vaccine have a promising neutralising profile with variable but interestingly high nAb titres, even with most variants, as described previously [27]. As recently observed, the new Delta variants are also neutralised by serum antibodies (sera which have variable antibody titres) that are “individual-dependent”, and these can be neutralised with lowered titres [27]. These data allow us to conclude that mRNA-based vaccine efficacy is even better than natural immunity in eliciting neutralising antibodies. In our study, we could not make the same conclusion for the AZD1222 vaccine, as we could not test more than two sera due to the long waiting period, which exceeded our study timeframe. However, we observed that neither the serum that had high IgG titres by CLIA nor the sera that had high neutralising profiles by seroneutralisation reacted with the 10 SARS-CoV-2 strains. Despite the promising viral neutralisation profiles of vaccinated individuals with the Pfizer/BioNTech vaccines in our cohort, a recent sero-epidemiological study showed that reinfection among patients previously infected by SARS-CoV-2 occurs at a lower rate (0.23%) than infection occurrence within previously vaccinated patients (5.1%) [38]. These findings are inconsistent with the outcomes obtained by the seroneutralisation tests, but viral neutralisation tests consist of in vitro approaches that may not reflect the effect of cellular immunity within the human body [39,40], as this technique is based exclusively on antibody–antigen interactions. A study published in May 2020 reported that during a COVID-19 infection, the SARS-CoV-2 spike (S) protein was found to be a nondominant target of the human CD8+ T cell response and that the recognition of the SARS-CoV-2 M (Matrix) antigen was similarly strong to the S antigen, which is unlike other coronaviruses [41]. Additionally, significant reactivity was found for other antigens, most notably the nsp6, ORF3a, and N antigens. Subsequently, COVID-19 vaccines that target only one antigen (the spike protein) will elicit a relatively narrow cellular response when compared with natural-induced T cells, which can target more than one antigen in convalescent patients [41,42]. Moreover, the large amounts of SARS-CoV-2 spike protein provided by mRNA- or adenovirus-based vaccines generally give rise to high titres of anti-spike circulating IgG [38]. As confirmed in a previous cohort performed in our institute, the SARS-CoV-2 spike protein is not the only immunogenicity marker for SARS-CoV-2 infection, as validated by automated western immunoblotting assays [43]. In naturally infected patients, lower IgG titres are detected, probably because the immunological antibody response involves primarily mucosal IgA. Our seroneutralisation data are thus consistent with this difference in the nAb titres between naturally infected and vaccinated individuals.

## Figures and Tables

**Table 1:** Tables showing the immuno-escape index (I-index), evaluating of the level of resistance of a SARS-CoV-2 variant to neutralising antibodies (nAb) directed against the RBD and the NTD of the spike protein. I-index is considered significant if >2. (wt = Original/B.1.1, mut = other).

Virus Strains	∆G RBD (LyCoV-555) kJ.mol^−1^	∆G NTD (4A8) kJ.mol^−1^	I-index (Immuno-Escape) ^1^	MNT ≥ 1/5 (nb/55)
Original/B.1.1	−244	−225	1.0	36/55 (65%)
Marseille-4/B.1.160	−245	−225	1.0	35/55 (64%)
B.1.214.2	−210	−225	1.1	39/55 (71%)
Alpha	−195	241	1.2	38/55 (69%)
Beta	−75	−118	2.6	21/55 (38%)
Gamma	−80	−82	2.9	34/55 (62%)
Marseille-484K.V1	−36	−258	3.0	28/55 (51%)
Marseille-501/A.27	−59	−114	3.1	34/55 (62%)
Delta/B.1.617.2	−68	−88	3.1	25/49 (51%)
Delta/AY.37	−68	−76	3.3	16/41 (39%)

^1^ I-index = 1/2 (ΔG_wt_/ΔG_mut_ (RBD-nAb) + ΔG_wt_/ΔG_mut_ (NTD-nAb)). The formula was designed so that the reference virus retrieved from PDB files 7KMG and 7C2 L had an I-index = 1. Under these conditions, the variants of the present studies could be classified into two groups: those with an I-index close to 1 (Marseille-4/B.1.160, B.1.214.2, and Alpha variants) that are predicted to be efficiently neutralised by natural and/or vaccinal nAbs and those with an I-index >2 (Beta, Marseille-484K.V1/R.1, Marseille-501/A.27, Gamma, Delta/B.1.617.2, and Delta/AY.37) that are likely to resist seroneutralisation.

## Supplementary Materials

The following are available online at https://www.mdpi.com/article/10.3390/v13112177/s1: Table S1. Serology of convalescent patients. Tables of data and serological testing results relative to all convalescent patients in the study.NA in CLIA results means no available serological test for the corresponding sera. Table S2. Serology of vaccinated participants. Tables of data and serological testing results relative to vaccinated participants in the study. Table S3. SARS CoV2 isolates lineage and spike mutations. Table extending related genomic information for each of the tested SARS-CoV-2 strains in our study. Clade, lineage, name of IHU isolate, and the corresponding nucleotide mutations, as well as amino acid mutations, are shown for each strain. The mean value for the nucleotide mutation between the variants is 44.2 mutations ± 15.760 SD. The minus sign (−) in the table means no mutation identified for the corresponding strain Table S4. Seroneutralisation results for convalescent patients. Table extending the neutralisation results of the 42 serum samples from the convalescent patient participating in our study as tested against 10 different strains of SARS-CoV-2. Monoclonal antibodies inbamlanivimab (LY-CoV 555) were used as controls. IgG titres tested by CLIA are presented in AU/mL. Seroneutralisation titre legend is shown on the right. Table S5. Seroneutralisation results for vaccinated participants. Table extending the neutralisation results of the 13 serum samples from the vaccinated participants in our study against 10 different strains of SARS-CoV-2. Monoclonal antibodies inbamlanivimab (LY-CoV 555) were used as controls. Seroneutralisation titre legends are shown on the right of each table.
