# Supplementary material for: High Individual Heterogeneity of Neutralizing Activities against the Original Strain and Nine Different Variants of SARS-CoV-2
Source: Viruses. 2021 Oct 28;13(11):2177. doi: 10.3390/v13112177 (PMC8623169; doi:10.3390/v13112177)
Supplement: Supplementary file 1 [file viruses-13-02177-s001.zip › Table S1.pdf]

Sera ID	Sex (F/M)	Year of birth
**I-1**	F	1969
**I-2**	F	1971
**I-3**	M	1960
**I-4**	M	1980
**I-5**	M	1982
**I-6**	F	1986
**I-7**	M	1943
**I-8**	M	1968
**I-9**	F	1965
**I-10**	M	1964
**I-11**	M	2006
**II-1**	F	1968
**II-2**	F	1995
**II-3**	F	1993
**II-4**	F	1975
**II-5**	M	1956
**II-6**	M	1935
**II-7**	M	1981
**II-8**	M	1952
**II-9**	F	2004
**UK-1**	M	1986
**UK-2**	M	1962
**UK-3**	M	1965
**UK-4**	F	1994
**UK-5**	M	1960
**UK-6**	F	1995
**UK-7**	M	1956
**UK-8**	F	1993
**UK-9**	M	1992
**UK-10**	F	1981
**SA-1**	F	1994
**SA-2**	M	1985
**SA-3**	F	1997
**SA-4**	F	1980
**SA-5**	M	1968
**SA-6**	F	1974
**SA-7**	M	1974
**SA-8**	F	1992
**SA-9**	F	1963
**SA-10**	F	1978
**SA-11**	F	1971
**SA-12**	F	1986

Age (years)	CLIA IgG (AU/mL)	IgG titer interpretation
52	NA	NA
50	193	Positive
61	169	Positive
41	190	Positive
39	162	Positive
35	47	Positive
78	400	Positive
53	>400	Positive
56	NA	NA
57	>400	Positive
15	197	Positive
53	15,8	Positive
26	35,5	Positive
28	76,2	Positive
46	43	Positive
65	45,7	Positive
86	30	Positive
40	32	Positive
69	161	Positive
17	25,4	Positive
35	15,8	Positive
59	123	Positive
56	86	Positive
27	20,3	Positive
61	221	Positive
26	32	Positive
65	147	Positive
28	42	Positive
29	31	Positive
40	10	Negative
27	13	Borderline
36	10	Negative
24	4	Negative
41	66,9	Positive
53	94	Positive
47	15	Borderline
47	34	Positive
29	11,7	Negative
58	19,2	Positive
43	26	Positive
50	39	Positive
35	31	Positive

Date of first positive PCR diagnosis	Date of sera collection
02/03/2020	16/06/2020
23/03/2020	06/03/2020
26/03/2020	15/06/2020
17/03/2020	19/04/2020
18/03/2020	27/05/2020
16/03/2020	22/05/2200
29/03/2020	20/04/2020
29/03/2020	27/04/2020
15/04/2020	05/07/2021
19/04/2020	05/11/2020
04/02/2020	05/07/2020
03/01/2021	04/01/2021
19/11/2020	19/04/2021
02/05/2021	19/04/2021
01/09/2021	15/04/2021
30/12/2020	02/12/2021
02/12/2021	03/08/2021
02/02/2021	05/05/2021
02/07/2021	03/03/2021
19/03/2021	21/04/2021
02/01/2021	04/08/2021
19/02/2021	19/03/2021
02/12/2021	17/03/2021
03/08/2021	15/04/2021
24/03/2021	19/04/2021
02/05/2021	23/04/2021
17/03/2021	26/04/2021
04/01/2021	15/05/2021
02/01/2021	18/05/2021
02/01/2021	05/03/2021
04/12/2021	05/02/2021
21/02/2021	30/04/2021
24/03/2021	20/04/2021
21/03/2021	16/04/2021
15/03/2021	06/07/2021
19/03/2021	06/07/2021
19/03/2021	06/07/2021
03/08/2021	06/08/2021
03/12/2021	06/08/2021
22/03/2021	06/08/2021
15/03/2021	06/08/2021
03/04/2021	06/08/2021

Time elapsed between 1st SARS-CoV-2-positive PCR test and sera collection
4 months
3 months
3 months
1 month
2 months
2 months
3 weeks
1 month
3 weeks
3 weeks
1 month
1 month
5 months
2 months
3 months
2 months
3 weeks
3 months
1 month
1 month
2 months
1 month
1 month
3 weeks
3 weeks
2 months
1 month
1 month
3 months
2 months
3 weeks
2 months
3 weeks
3 weeks
3 months
3 months
3 months
3 months
3 months
3 months
3 months
3 months
