# Supplementary material for: High Individual Heterogeneity of Neutralizing Activities against the Original Strain and Nine Different Variants of SARS-CoV-2
Source: Viruses. 2021 Oct 28;13(11):2177. doi: 10.3390/v13112177 (PMC8623169; doi:10.3390/v13112177)
Supplement: Supplementary file 1 [file viruses-13-02177-s001.zip › Table S2.pdf]

Sera ID	Sex (F/M)	Year of birth	Age (years)
**V-Pfizer-1**	M	1967	54
**V-Pfizer-2**	M	1961	60
**V-Pfizer-3**	F	1969	52
**V-Pfizer-4**	M	1952	69
**V-Pfizer-5**	F	1969	52
**V-Pfizer-6**	F	1994	27
**V-Pfizer-7**	M	1992	29
**V-Pfizer-8**	F	1993	28
**V-Pfizer-9**	M	1992	29
**V-Pfizer-10**	M	1939	82
**V-Pfizer-11**	M	1992	29
**Astra-1**	F	1963	58
**Astra-2**	M	1963	58

CLIA IgG (AU/mL)	gG titer interpretatio	Vaccine type	accine Dosage uptak
>400	Positive	Pfizer/BioNTech	2 doses
300	Positive	Pfizer/BioNTech	2 doses
>400	Positive	Pfizer/BioNTech	2 doses
192	Positive	Pfizer/BioNTech	2 doses
152	Positive	Pfizer/BioNTech	2 doses
>400	Positive	Pfizer/BioNTech	2 doses
229	Positive	Pfizer/BioNTech	2 doses
262	Positive	Pfizer/BioNTech	2 doses
369	Positive	Pfizer/BioNTech	2 doses
3,5	Negative	Pfizer/BioNTech	2 doses
363	Positive	Pfizer/BioNTech	2 doses
84,7	Positive	Vaxzevria AZD1222	2 doses
277	Positive	Vaxzevria AZD1222	2 doses

Date of last vaccine injection	Date of sera collection
02/01/2021	15/02/2021
03/09/2021	17/03/2021
03/09/2021	18/03/2021
02/05/2021	22/03/2021
27/01/2021	04/08/2021
04/08/2021	22/04/2021
17/02/2021	19/04/2021
02/06/2021	22/04/2021
02/10/2021	19/04/2021
03/01/2021	04/01/2021
22/02/2021	19/04/2021
04/03/2021	05/03/2021
05/12/2021	31/05/2021

Time between last vaccine injection and sera collection
2 weeks
10 days
10 days
1 month
3 months
12 days
2 months
2 months
2 months
1 month
2 months
1 month
2 weeks
