# Supplementary material for: High Individual Heterogeneity of Neutralizing Activities against the Original Strain and Nine Different Variants of SARS-CoV-2
Source: Viruses. 2021 Oct 28;13(11):2177. doi: 10.3390/v13112177 (PMC8623169; doi:10.3390/v13112177)
Supplement: Supplementary file 1 [file viruses-13-02177-s001.zip › Table S3.pdf]

Strain Name	PANGO lineage	Clade	IHU isolate	Total number of nucleotide substitutions	Total number of nucleotide deletions	Total number of nucleotide insertions	Total number of amino acid substitutions	Total number of amino acid deletions	Nucleotide substitutions	Nucleotide deletions	Nucleotide insertions
**Original/B.1.1 Virus**	**B.1.1**	**20B**	**IHUMI717**	19	9	0	11	3	C241T, C313T, C3037T, C4755T, A6488G, A7903G, C14408T, C15024T, G19518T, T21318C, A23403G, G23607C, C24700T, G26143T, C26333T, G28845T, G28881A, G28882A, G28883C	3341-3349	-
**Marseille-4/B.1.160 variant**	**B.1.160**	**20A**	**IHUMI2096**	20	0	0	11	0	C241T, C3037T, C4543T, G5629T, G9526T, C11497T, G13993T, C14408T, G15766T, A16889G, G17019T, C18877T, G22992A, A23403G, G25563T, C25710T, C26735T, T26876C, G28975C, G29399A	-	-
**Marseille-501/A.27 variant**	**A.27**	**19B**	**IHUMI3217**	31	15	0	20	4	A361G, C1122T, C2509T, A7112G, T7999C, C8782T, A9204G, A11217G, C15103G, C16293T, C16466T, G17815A, A18366G, G19925A, A20262G, C21614T, T22917G, A23063T, C23520T, C23525T, G23948T, G24089A, G25218T, G25494T, T25541C, C27247T, T27499C, T28144C, A28273T, G28878A, C29149A	26161-26168, 27388, 28248-28253	-
**Marseille-484K.V1/R.1variant**	**R.1**	**20B**	**IHUMI3239**	24	1	0	14	0	C241T, C3037T, C13860T, C14220A, C14340T, C14408T, C14599T, G17551A, C18877T, A19167G, C19274A, T19839C, G22017T, G23012A, A23403G, G23868T, T26604C, C27213T, T28334C, C28833T, G28881A, G28882A, G28883C, G29527T	28271	-
**B.1.214.2variant**	**B.1.214.2**	**20B**	**IHUMI3246**	30	32	10	28	11	C241T, G587A, G1394T, C3037T, A4457G, C4891T, C5184T, C5907T, A9204G, A11430G, C11941T, A12077T, C12312T, C14408T, A15187G, C15324T, C19975A, C22802A, G22899A, T22912G, A23403G, C23709T, T24071G, T27576C, C27881A, G28280C, A28281T, T28282A, C28846T, C28887T	25447-25476, 28271-28272	6698:T, 22206:ACAGATCGA
**Alpha variant**	**B.1.1.7**	**20I (Alpha, V1)**	**IHUMI3076**	31	25	0	20	8	C241T, C913T, A1399G, C3037T, C3267T, C5388A, C5986T, T6954C, C14408T, C14676T, C15279T, T16176C, A17615G, A23063T, C23271A, A23403G, C23604A, C23709T, T24506G, G24914C, G26730C, C27972T, G28048T, A28111G, G28280C, A28281T, T28282A, G28881A, G28882A, G28883C, C28977T	11288-11296, 12041-12046, 21765-21770, 21992-21994, 28271	-
**Beta variant**	**B.1.351.2**	**20H (Beta, V2)**	**IHUMI3147**	27	19	0	18	6	C28T, G174T, C241T, C1059T, A2692T, C3037T, G5230T, A8052G, A10323G, G10396T, C10632T, C14408T, C21614T, A21801C, A22206G, G22813T, G23012A, A23063T, A23403G, T23560C, C23664T, G25563T, C25904T, C26456T, C28253T, T28729C, C28887T	26, 11288-11296, 22283-22291	-
**Gamma variant**	**P.1**	**20J (Gamma, V3)**	**IHUMI3191**	36	10	5	23	3	C28T, C241T, T733C, C2749T, C3037T, C3828T, A5648C, A6319G, A6613G, C12778T, C13860T, C14408T, G17259T, C21614T, C21621A, C21638T, G21974T, G22132T, A22812C, G23012A, A23063T, A23403G, C23525T, C23606T, C24642T, G25088T, T26149C, G28167A, C28512G, A28877T, G28878C, G28881A, G28882A, G28883C, T29834A, T29858A	26, 11288-11296	5:C, 28264:AACA
**Delta/B.1.617.2 variant**	**B.1.617.2**	**21J (Delta)**	**IHUMI3396**	38	13	0	31	4	G210T, C241T, C3037T, G4181T, C6402T, C7124T, C8986T, G9053T, C9559T, C10029T, G11083T, A11201G, A11332G, C14408T, C15240T, G15451A, C16466T, C19220T, C21618G, C21762T, C21846T, T22917G, C22995A, A23403G, C23604G, G23607A, C25469T, T26767C, G27358T, T27638C, C27752T, C27874T, T28007C, A28461G, G28881T, G28916T, G29402T, G29742T	22029-22034, 28248-28253, 28271	-
**Delta/AY.37 variant**	**AY.37**	**21I (Delta)**	**IHUMI3630**	34	13	0	28	4	G210T, C241T, C2509T, C3037T, C5184T, A5584G, C9891T, T11418C, C11514T, C13019T, C14408T, G15451A, C16466T, C16726T, A18931G, C19118T, C21618G, C22227T, T22917G, C22995A, A23403G, C23604G, C23606T, G24410A, C25317T, C25469T, C26753T, T26767C, T27638C, C27752T, A28461G, G28881T, G29402T, G29742T	22029-22034, 28248-28253, 28271	-

Strain Name	PANGO lineage	Clade	IHU isolate	Amino acid substitutions	Spike amino acid subsitutions	Amino acid deletions	Spike amino acid deletions	Amino acid substitutions reported as associated with immune escape and/or modified infectivity/transmissibility *
**Original/B.1.1 Virus**	**B.1.1**	**20B**	**IHUMI717**	E:T30I, N:R191L, N:R203K, N:G204R, ORF1a:P1497L, ORF1a:I2075V, ORF1b:P314L, ORF1b:L2017F, ORF3a:G251C, S:D614G, S:R682P	D614G, R682P	ORF1a:N1026-, ORF1a:S1027-, ORF1a:F1028-	-	D614G
**Marseille-4/B.1.160 variant**	**B.1.160**	**20A**	**IHUMI2096**	N:M234I, N:A376T, ORF1a:M3087I, ORF1b:A176S, ORF1b:P314L, ORF1b:V767L, ORF1b:K1141R, ORF1b:E1184D, ORF3a:Q57H, S:S477N, S:D614G	S477N, D614G	-	-	S477N, D614G
**Marseille-501/A.27 variant**	**A.27**	**19B**	**IHUMI3217**	N:S202N, ORF1a:P286L, ORF1a:T2283A, ORF1a:D2980G, ORF1a:N3651S, ORF1b:R546G, ORF1b:P1000L, ORF1b:G1450R, ORF1b:C2153Y, ORF3a:V50A, ORF7a:S36P, ORF8:L84S, S:L18F, S:L452R, S:N501Y, S:A653V, S:H655Y, S:D796Y, S:D843N, S:G1219V	L18F, L452R, N501Y, A653V, H655Y, D796Y, D843N, G1219V	ORF3a:N257-, ORF3a:P258-, ORF8:D119-, ORF8:F120-	-	L452R, N501Y, L18F
**Marseille-484K.V1/R.1variant**	**R.1**	**20B**	**IHUMI3239**	M:F28L, N:S21P, N:S187L, N:R203K, N:G204R, N:Q418H, ORF1b:D251E, ORF1b:P314L, ORF1b:G1362R, ORF1b:P1936H, S:W152L, S:E484K, S:D614G, S:G769V	W152L, E484K, D614G, G769V	-	-	E484K, D614G
**B.1.214.2variant**	**B.1.214.2**	**20B**	**IHUMI3246**	N:D3L, N:T205I, ORF1a:V108I, ORF1a:E377*, ORF1a:I1398V, ORF1a:P1640L, ORF1a:T1881I, ORF1b:P314L, ORF1b:R574G, ORF1b:L2170I, ORF3a:R6D, ORF3a:I7L, ORF3a:T9R, ORF3a:G11F, ORF3a:K21G, ORF3a:D22T, ORF3a:A23V, ORF3a:P25L, ORF3a:S26K, ORF3a:D27Q, ORF3a:F28G, ORF7b:H42Q, S:Q414K, S:G446D, S:N450K, S:D614G, S:T716I, S:Y837D	Q414K, G446D, N450K, D614G, T716I, Y837D	ORF3a:M1-, ORF3a:D2-, ORF3a:L3-, ORF3a:F4-, ORF3a:V13-, ORF3a:T14-, ORF3a:L15-, ORF3a:K16-, ORF3a:Q17-, ORF3a:G18-, ORF3a:E19-	-	D614G
**Alpha variant**	**B.1.1.7**	**20I (Alpha, V1)**	**IHUMI3076**	M:V70L, N:D3L, N:R203K, N:G204R, N:S235F, ORF1a:T1001I, ORF1a:A1708D, ORF1a:I2230T, ORF1b:P314L, ORF1b:K1383R, ORF8:Q27*, ORF8:R52I, ORF8:Y73C, S:N501Y, S:A570D, S:D614G, S:P681H, S:T716I, S:S982A, S:D1118H	N501Y, A570D, D614G, P681H, T716I, S982A, D1118H	ORF1a:S3675-, ORF1a:G3676-, ORF1a:F3677-, ORF1a:D3926-, ORF1a:I3927-, S:H69-, S:V70-, S:Y144-	H69-, V70-, Y144-	Δ69–70, Δ144, D614G, N501Y
**Beta variant**	**B.1.351.2**	**20H (Beta, V2)**	**IHUMI3147**	E:P71L, N:T205I, ORF1a:T265I, ORF1a:K1655N, ORF1a:N2596S, ORF1a:K3353R, ORF1a:A3456V, ORF1b:P314L, ORF3a:Q57H, ORF3a:S171L, S:L18F, S:D80A, S:D215G, S:K417N, S:E484K, S:N501Y, S:D614G, S:A701V	L18F, D80A, D215G, L242H, K417N, E484K, N501Y, D614G, A701V	ORF1a:S3675-, ORF1a:G3676-, ORF1a:F3677-, S:L241-, S:L242-, S:A243-	A243-, L244-, H245-	E484K, Δ243, D614G, N501Y, K417N, L18F
**Gamma variant**	**P.1**	**20J (Gamma, V3)**	**IHUMI3191**	N:P80R, N:R203K, N:G204R, ORF1a:S1188L, ORF1a:K1795Q, ORF1b:P314L, ORF1b:E1264D, ORF3a:S253P, ORF8:E92K, ORF9b:Q77E, S:L18F, S:T20N, S:P26S, S:D138Y, S:R190S, S:K417T, S:E484K, S:N501Y, S:D614G, S:H655Y, S:R682W, S:T1027I, S:V1176F	L18F, T20N, P26S, D138Y, R190S, K417T, E484K, N501Y, D614G, H655Y, R682W, T1027I, V1176F	ORF1a:S3675-, ORF1a:G3676-, ORF1a:F3677-	-	E484K, D614G, N501Y, K417T, L18F, T20N
**Delta/B.1.617.2 variant**	**B.1.617.2**	**21J (Delta)**	**IHUMI3396**	M:I82T, N:D63G, N:R203M, N:G215C, N:D377Y, ORF1a:A1306S, ORF1a:P2046L, ORF1a:P2287S, ORF1a:V2930L, ORF1a:T3255I, ORF1a:L3606F, ORF1a:T3646A, ORF1b:P314L, ORF1b:G662S, ORF1b:P1000L, ORF1b:A1918V, ORF3a:S26L, ORF6:D53Y, ORF7a:V82A, ORF7a:T120I, ORF7b:T40I, ORF9b:T60A, S:T19R, S:A67V, S:T95I, S:R158G, S:L452R, S:T478K, S:D614G, S:P681R, S:R682Q	T19R, A67V, T95I, R158G, L452R, T478K, D614G, P681R, R682Q	ORF8:D119-, ORF8:F120-, S:E156-, S:F157-	E156-, F157-	L452R, D614G
**Delta/AY.37 variant**	**AY.37**	**21I (Delta)**	**IHUMI3630**	M:I82T, N:D63G, N:R203M, N:D377Y, ORF1a:P1640L, ORF1a:A3209V, ORF1a:V3718A, ORF1a:T3750I, ORF1b:P314L, ORF1b:G662S, ORF1b:P1000L, ORF1b:H1087Y, ORF1b:I1822V, ORF1b:A1884V, ORF3a:S26L, ORF7a:V82A, ORF7a:T120I, ORF9b:T60A, S:T19R, S:R158G, S:A222V, S:L452R, S:T478K, S:D614G, S:P681R, S:R682W, S:D950N, S:S1252F	T19R, R158G, A222V, L452R, T478K, D614G, P681R, R682W, D950N, S1252F	ORF8:D119-, ORF8:F120-, S:E156-, S:F157-	E156-, F157-	L452R, A222V, D614G

* According to: Tao K, Tzou PL, Nouhin J, Gupta RK, de Oliveira T, Kosakovsky Pond SL, Fera D, Shafer RW. The biological and clinical significance of emerging SARS-CoV-2 variants. Nat Rev Genet. 2021 Sep 17:1–17. doi: 10.1038/s41576-021-00408-x. Epub ahead of print. PMID: 34535792; PMCID: PMC8447121.
