# Supplementary material for: High Individual Heterogeneity of Neutralizing Activities against the Original Strain and Nine Different Variants of SARS-CoV-2
Source: Viruses. 2021 Oct 28;13(11):2177. doi: 10.3390/v13112177 (PMC8623169; doi:10.3390/v13112177)
Supplement: Supplementary file 1 [file viruses-13-02177-s001.zip › Table S4.pdf]

Ab Category	Sample ID	IgG (AU/mL)	Original/B.1.1	Marseille-4/ B.1.160	Alpha/B.1.1.7	Marseille-501/ A.27	Beta/B.1.351.2	Gamma/P.1	Marseille-484K.V1/R.1	B.1.214.2	Delta/B.1.617.2	Delta/AY.37
mAbs	LY-CoV555	35 000 µg/mL	0.224 µg/mL	0.224 µg/mL	1.120 µg/mL	3500 µg/mL				1.120 µg/mL	3500 µg/mL	
Patients infected with Original virus	I-1	NA	1/10	1/10	1/10	1/10		1/10	1/20	1/40		
	I-2	193	1/40	1/20	1/20	1/80		1/10		1/40	1/20	NA
	I-3	169	1/10	1/10	1/20	1/40		1/20	1/40	1/40	NA	NA
	I-4	190	1/40		1/10	1/20		1/20	1/40	1/40	1/40	NA
	I-5	162	1/10	1/10	1/10	1/10		1/10	1/10	1/20	NA	NA
	I-6	47		1/10						1/10	NA	NA
	I-7	400	1/10	1/10	1/10	1/5		1/5		1/10	NA	NA
	I-8	>400	1/80	1/80	1/80	1/40	1/80	1/160	1/80	1/80	NA	NA
	I-9	NA		1/20	1/10	1/20	1/5	1/20	1/10	1/10	NA	NA
	I-10	>400	1/40	1/20	1/10	1/40	1/20	1/20	1/40	1/80	1/40	NA
	I-11	197	1/40	1/10	1/10					1/20		NA
Patients infected with Marseille-4 strain	II-1	15.8										
	II-2	35.5	1/5	1/5		1/5		1/5		1/5		
	II-3	76.2	1/20	1/20	1/20	1/20	1/5	1/5	1/5	1/10	1/5	1/5
	II-4	43	1/5	1/5						1/5		
	II-5	45.7	1/10								1/20	
	II-6	30										NA
	II-7	32			1/5				1/5	1/5		
	II-8	161	1/40	1/20	1/40	1/10		1/10	1/5	1/80	1/20	NA
	II-9	25.4										NA
Patients infected with Alpha strain	UK-1	15.8	1/20		1/10	1/40	1/20	1/40	1/10	1/20	1/5	1/5
	UK-2	123	1/5	1/5	1/10	1/10		1/10	1/5	1/10	1/10	1/5
	UK-3	86	1/5		1/5	1/5				1/5		
	UK-4	20.3	1/5		1/10							
	UK-5	221	1/20	1/40	1/160	1/20	1/40	1/40	1/80	1/80	1/20	1/20
	UK-6	32	1/10	1/5	1/20	1/5	1/5	1/5	1/5	1/10	1/10	
	UK-7	147	1/10	1/5	1/20	1/10	1/5	1/5	1/5	1/10	1/10	1/5
	UK-8	42	1/10	1/20	1/20	1/5		1/5		1/5	1/5	
	UK-9	31	1/10	1/20	1/20	1/10				1/5	1/10	
	UK-10	10			1/5							
Patients infected with the Beta strain	SA-1	13										NA
	SA-2	10										
	SA-3	4										
	SA-4	66.7			1/20	1/10	1/40	1/40	1/5	1/40		
	SA-5	94	1/5	1/10	1/20	1/10	1/10	1/10	1/10	1/20	1/10	1/5
	SA-6	15					1/5	1/5				
	SA-7	34		1/5	1/5		1/5	1/5				
	SA-8	11.7										
	SA-9	19.2					1/5					
	SA-10	26			1/5		1/10	1/10	1/5			
	SA-11	39						1/10				
	SA-12	31					1/5	1/5				

Not available: NA

No neutralization

Highest serum neutralizing dilution: 1/160, 1/80, 1/40, 1/20, 1/10, 1/5

Bamlanivimab LY-Cov-555 Concentrations (µg/ml): 0.224, 1.12, 5.6, 28, 140, 700, 3500
