# Supplementary material for: High Individual Heterogeneity of Neutralizing Activities against the Original Strain and Nine Different Variants of SARS-CoV-2
Source: Viruses. 2021 Oct 28;13(11):2177. doi: 10.3390/v13112177 (PMC8623169; doi:10.3390/v13112177)
Supplement: Supplementary file 1 [file viruses-13-02177-s001.zip › Table S5.pdf]

Ab Category	Sample ID	IgG (AU/mL)	Original/B.1.1	Marseille-4/ B.1.160	Alpha/B.1.1.7	Marseille-501/ A.27	Beta/B.1.351.2	Gamma/P.1	Marseille-484K.V1/R.1	B.1.214.2	Delta/B.1.617.2	Delta/AY.37
mAbs	LY-CoV555	35 000 µg/mL	0.224 µg/mL	0.224 µg/mL	1.120 µg/mL	3500 µg/mL				1.120 µg/mL	3500 µg/mL	
mRNA-based Pfizer/ BioNTech vaccine	V-Pfizer-1	>400	1/80	1/80	1/80	1/80	1/20	1/80	1/80	1/160	1/80	1/80
mRNA-based Pfizer/ BioNTech vaccine	V-Pfizer-2	300	1/5	1/5	1/10	1/10		1/5	1/10	1/20	1/10	1/10
mRNA-based Pfizer/ BioNTech vaccine	V-Pfizer-3	>400	1/10	1/5	1/10	1/20		1/5	1/10	1/80	1/10	1/10
mRNA-based Pfizer/ BioNTech vaccine	V-Pfizer-4	192	1/5	1/5	1/5	1/5			1/5	1/10		
mRNA-based Pfizer/ BioNTech vaccine	V-Pfizer-5	152	1/40	1/10	1/5	1/10		1/20	1/20	1/40	1/10	1/5
mRNA-based Pfizer/ BioNTech vaccine	V-Pfizer-6	>400	1/80	1/160	1/80	1/40	1/40	1/80	1/40	1/40	1/80	1/80
mRNA-based Pfizer/ BioNTech vaccine	V-Pfizer-7	229	1/40	1/40	1/20	1/20	1/5	1/20	1/20	1/40	1/10	1/10
mRNA-based Pfizer/ BioNTech vaccine	V-Pfizer-8	262	1/80	1/80	1/80	1/80	1/20	1/80	1/40	1/80	1/40	1/40
mRNA-based Pfizer/ BioNTech vaccine	V-Pizer-9	369	1/40	1/40	1/40	1/40	1/20	1/160	1/160	1/80	1/40	1/20
mRNA-based Pfizer/ BioNTech vaccine	V-Pfizer-10	3.5										
mRNA-based Pfizer/ BioNTech vaccine	V-Pfizer-11	363	1/80	1/40	1/40	1/40	1/20	1/40	1/40	1/40	1/40	1/20
Adenovirus-based AZD1222	Astra-1	84.7	1/5	1/5		1/5				1/5		
Adenovirus-based AZD1222	Astra-2	277	1/20	1/10	1/10	1/20	1/5	1/20	1/5	1/40	1/10	1/5

No neutralization

Highest serum neutralizing dilution

1/160

1/80

1/40

1/20

1/10

1/5

Bamlanivimab LY-Cov-555 Concentrations (µg/ml)

0.224

1.12

5.6

28

140

700

3500
